# Improving the Quality and Utility of Electronic Health Record Data through Ontologies

**DOI:** 10.3390/standards3030023

**Published:** 2023-09-15

**Authors:** Asiyah Yu Lin, Sivaram Arabandi, Thomas Beale, William D. Duncan, Amanda Hicks, William R. Hogan, Mark Jensen, Ross Koppel, Catalina Martínez-Costa, Øystein Nytrø, Jihad S. Obeid, Jose Parente de Oliveira, Alan Ruttenberg, Selja Seppälä, Barry Smith, Dagobert Soergel, Jie Zheng, Stefan Schulz

**Affiliations:** 1 National Institutes of Health, Bethesda, MD 20892, USA; 2 ONTOPRO, Houston, TX 77025, USA; 3 Ars Semantica Ltd., London W4 1PQ, UK; 4 College of Dentistry, University of Florida, Gainesville, FL 32610, USA; 5 The Johns Hopkins University Applied Physics Laboratory, Laurel, MD 20723, USA; 6 Data Science Institute, Medical College of Wisconsin, Milwaukee, WI 53226, USA; 7 CUBRC Inc., Buffalo, NY 14225, USA; 8 Department of Medical Informatics, Jacobs School of Medicine, University at Buffalo, Buffalo, NY 14260, USA; 9 Department of Medical Informatics, School of Medicine, University of Pennsylvania, Philadelphia, PA 19104, USA; 10 Department of Informatics and Systems, Faculty of Computer Science, University of Murcia, 30100 Murcia, Spain; 11 Department of Computer Science, UIT Arctic University of Norway, 9037 Tromsø, Norway; 12 Department of Computer Science, Norwegian University of Science and Technology, 7491 Trondheim, Norway; 13 Department of Public Health Sciences, Medical University of South Carolina, Charleston, SC 29425, USA; 14 Aeronautics Institute of Technology, São José dos Campos 12228-900, Brazil; 15 School of Dental Medicine, University at Buffalo, Buffalo, NY 14260, USA; 16 Department of Business Information Systems, University College Cork, T12 K8AF Cork, Ireland; 17 Department of Philosophy, University at Buffalo, Buffalo, NY 14260, USA; 18 Unit for Laboratory Animal Medicine, University of Michigan Medical School, Ann Arbor, MI 48104, USA; 19 Institute for Medical Informatics, Statistics and Documentation, Medical University of Graz, 8036 Graz, Austria; 20 Averbis GmbH, Salzstrasse 15, 79098 Freiburg im Breisgau, Germany

**Keywords:** electronic health record, EHR, ontology, semantics, interoperability, clinical informatics, biomedical informatics, standards, terminology, information model

## Abstract

The translational research community, in general, and the Clinical and Translational Science Awards (CTSA) community, in particular, share the vision of repurposing EHRs for research that will improve the quality of clinical practice. Many members of these communities are also aware that electronic health records (EHRs) suffer limitations of data becoming poorly structured, biased, and unusable out of original context. This creates obstacles to the continuity of care, utility, quality improvement, and translational research. Analogous limitations to sharing objective data in other areas of the natural sciences have been successfully overcome by developing and using common ontologies. This White Paper presents the authors’ rationale for the use of ontologies with computable semantics for the improvement of clinical data quality and EHR usability formulated for researchers with a stake in clinical and translational science and who are advocates for the use of information technology in medicine but at the same time are concerned by current major shortfalls. This White Paper outlines pitfalls, opportunities, and solutions and recommends increased investment in research and development of ontologies with computable semantics for a new generation of EHRs.

## 1. Purpose of This Document

This Perspective synthesizes the presentations and discussions of the Fifth Annual Workshop [1] of the Clinical and Translational Science Ontology Group, held in Buffalo, NY, USA, 7–8 September 2016, and the continuous discussion that followed until 2019. The Clinical and Translational Science Award (CTSA) hubs are funded by the National Institutes of Health’s National Center for Advancing Translational Science (NCATS) with a focus on accelerating translational research and includes a consortium of approximately 60 centers throughout the U.S. The authors attempt to show in what follows how the quality and utility of electronic health record (EHR) data can benefit from a general strategy that emphasizes the use of formal, well-structured, consensus-based clinical terminologies and ontologies. These benefits include the following:

- The creation of EHR data, which are more accurate, represent patient and care-related phenomena with greater precision and faithfulness, and are more effectively computable;
- The support of user interfaces that facilitate standard data entry support dynamic data checking and provide improved data retrieval and data analysis, thus warranting a closer match (i) between what the user intends to record and what was actually recorded and (ii) between the user’s information need and its fulfillment;
- The improvement of interoperability of healthcare systems, thus providing a more comprehensive body of clinical data from heterogeneous sources that can not only support clinical decisions, improve quality of care, and avoid medical errors but also enable more advanced digitally based testing of clinical and translational research hypotheses.

This Perspective will assemble evidence to support these claims. It will also identify problems standing in the way of the effective use of an ontological approach in the EHR domain. Its main purposes are as follows:

- To educate all research and healthcare communities about what can be carried out to enhance and improve the value and usability of EHRs with a particular focus on translational research.
- To educate the following communities about what can be carried out to improve the quality and utility of clinical data by enhancing the usefulness of EHRs (and also using parallel strategies, genomic and other clinically relevant data) at the point of care without increasing the data and documentation burden:
  - Designers and administrators of EHRs;
  - Designers and administrators of Clinical Trial Management Systems;
  - Communities concerned with setting standards for healthcare data;
  - Researchers who conduct clinical studies using EHR data;
  - Health system leadership of CTSA hubs;
  - CTSA principal investigators;
  - National Center for Advancing Translational Sciences (NCATS) staff;
  - Members of government agencies with a stake in making available and using quality clinical data for translational research.
- To lay out opportunities for meaningful next steps, especially as they concern coordination among CTSA hubs.
- To provide examples of what has already been achieved.

The paper focuses on the quality of clinical data (see Box 1), including quality-related aspects of data entry and data access. It does not address issues of data security, privacy, and ethics around the EHR, nor does it address issues of performance or specific implementations.

## 2. Introduction

Our current methods for advancing and organizing the information gained in the course of clinical care and through biomedical research are the outcomes of an evolutionary process in which generations of clinicians, researchers, and persons with information technology expertise have participated. This is also true for the conventions governing the communication of these results through different types of unstructured or semi-structured documents and information artifacts in EHRs and other information systems. Human cognitive abilities are increasingly challenged by the sheer amount and variety of data that need to be processed. The specific concerns addressed here derive from the fact that matters are made more difficult in the healthcare domain by deficiencies in the usability of most healthcare information technology. We believe that far too little work has been carried out to address the shortfalls in usability and to focus on the human side of healthcare data entry and access. At the same time, we recognize the gains achieved through the use of ever more powerful computer technology to support data and knowledge management for clinical and research data compared with traditional, paper-based documentation. In the U.S., the American Dental Association (ADA) and the Centers for Medicare & Medicaid Services (CMS) have spearheaded the introduction of EHR systems. In addition, the Patient Protection and Affordable Care Act (ACA [5]), now the 21st Century Cure Act (Cures Act), signed into law on 13 December 2016, made sharing electronic health information the expected norm in healthcare. Whereas the ACA sought to justify this measure in terms of assumed advances in the quality of care and cost reductions, the hasty and often disruptive introduction of commercial EHR technology has caused well-known problems for many clinical communities. Our task here is to document certain less well-known problems concerning the reuse of EHR data for the purpose of research, known as real-world data (RWD) for research.

### 2.1. Problem Statement

Table 1 provides an overview of the standard and advanced purposes of EHRs, together with technical requirements. Given current EHR implementations and scenarios of use, we identify three major obstacles to realizing the full potential of EHRs:

1. There is a failure of human-system interoperability deriving from the fact that the current layout and functionality of digital tools not only create a barrier to fulfilling routine documentation and communication but also lead to poor documentation and require clinicians to spend more time to correct or improve the documentation than expected.
2. As cumbersome computer interfaces have taken the place of established modes of communicating and documenting based on paper, phone, and fax to a degree, they bear the risk of increasingly replacing the patient as the principal object of focus during a clinical encounter. As a result, a clinician spends less time with the patients.
3. There is a failure of the system–system interoperability—initially advocated as a main rationale for the introduction of EHR technology—however, in reality, any given EHR system is often not even interoperable with the computer systems across the same enterprise.

To those aware of the shortfalls along the dimensions listed in Table 1, it is not surprising that most postulated benefits of EHRs have not been significantly realized. These include the improvement of patient safety, clinical decision support, clinical cost reduction, clinical quality assessment, disease reporting, cohort building, recruitment for clinical trials, comparative effectiveness research, pragmatic trials, predictive analytics, and many more.

Prospects for clinical data use and reuse for research purposes still need to be substantiated. An example of reuse (also known as secondary use [6]) is the extraction of structured content usable for research from the body of EHR data to enable large-scale clinical phenotyping for translational research—where problems such as local implementations of EHRs and the predominance of unstructured text data within EHRs hampered the realization of initial enthusiasm. Some progress has been made in showing how EHR data can be used to answer clinical questions [7], but progress has lagged expectations. To achieve the sorts of advances originally promised, EHR systems need to be reformed.

These reforms need to be carried out to enable EHR data to be used more effectively for research without neglecting multiple efforts needed to improve the efficacy and quality of care by enhanced usability in primary EHR use.

Further deficiencies of EHR data that hinder their reuse in translational research scenarios are summarized in Table 2 [8].

These caveats, which were formulated specifically in the context of comparative effectiveness research (CER), need to be supplemented by numerous known idiosyncrasies in EHR systems, which frustrate data entry and affect data reuse not only for research but also for everyday purposes (see Box 2).

There is an abundance of types of poor or poorly implemented standards. The old maxim “garbage in, garbage out” therefore applies not only—nor even primarily—to users of EHRs, but primarily to their designers and developers. By placing a nonsense value in a dropdown list, the EHR developer or implementer is supporting “garbage in”. The “garbage out”, i.e., bad data received by providers, patients, researchers, and health systems, result in frustrated end users and a loss of trust in the EHR’s reliability and sensibility. This garbage hinders EHR data reuse for translational research due to inaccurate and incomplete data, a justified lack of trust, and multiple different sorts of failures of alignment.

In current EHR implementations, misspellings in free text entries do not trigger correction suggestions, a standard functionality in mobile devices and web browsers. Physicians who move from one hospital to another using the same EHR product from the same vendor report that although the systems were related (like speaking ‘Spanish and Italian’), the data and interfaces differed enough that assumptions of similarities could be treacherous [9]. Presurgical questionnaires mindlessly ask both male and female patients the same sex-specific questions. What happens when an adolescent male facetiously answers “yes” to the question of whether his teeth hurt more during his period? A human can discount his answer, but can the computer? After all, it did not know enough not to ask the question in the first place.

Given that the cost of the EHR for a large academic health center can be as high as $1.2 billion [10], should we not expect a baseline of common sense in EHR schemas, screens, and the data they generate?

### 2.2. The Role of Ontologies with Computable Semantics for the Improvement of Clinical Data Quality

The solutions to these kinds of problems are multifaceted and need to address the whole lifecycle of clinical data. However, they all touch upon three aspects of clinical data: their content, structure, and semantics. An optimal quality of clinical data requires that the meaning of all data elements be described in a way that is not only unambiguous and understandable for humans but also processable by machines. This desideratum should be fulfilled by computable representations that provide universally valid descriptions of the entities of a domain, together with the way they are connected. These representations, commonly known as ontologies or, if enriched by computable semantics, also as formal ontologies [11], have been increasingly recognized as fundamental resources for knowledge management. Together with other resources and technologies such as terminologies and human language technologies based on natural language processing (NLP), ontologies are supposed to inform these three aspects more intelligently than ad hoc approaches by EHR vendors and standards development organizations who often focus more on interoperability between systems than on content and meaning that is in systems. In the following, we provide a list of desiderata for clinical data management within an ontology-based framework:

- Data acquisition, including data processing and storage, is constantly supported by appropriate terminology linked to an ontology-based semantic layer, clinical processes, and users. From this layer, we should expect the following:
  - Personalized interfaces that minimize the number of actions required for a given task.
  - Intuitive guidance at data entry, which includes the detection of redundant or erroneous entries.
  - Context-sensitivity that ensures that only the data needed in a given scenario are, in fact, provided by the system.
  - Adjustment of the system to the user’s communication behavior, including the sublanguages used.
  - Understanding of semistructured free text and voice input.
  - Semantic error-detection and alerting approaches [12].
- Supported and enriched by the semantic layer, normalized content ironing out the variability of data input. This requires the following:
  - Information to be stored in a standardized and ontology-aware way.
  - Addressing the requirements for structured information use and reuse for a variety of use cases.
  - Flexibility by ensuring that data mimic as far as possible the structure of reality.
  - Constant monitoring of all actions, with log data feeding a learning system (see Box 3), which aims at optimizing processes and underlying resources.
  - Explicitness of each data element regarding its context and provenance.
  - Data reuse enabled by ontology-based data query should address the following aspects:
  - User-friendly, self-explaining query interfaces, which facilitate semantic cross-linkage of patient-related information with general knowledge such as clinical guidelines.
  - Push and pull scenarios that address the information needs of different user groups (clinicians, researchers, administrators, data managers, and patients) and data management tasks need to be supported.
  - Improved reuse of data for research, which includes the interoperation of hitherto separated resources, e.g., EHR with clinical trial management and data capture systems, as well as electronic case report forms.
  - Powerful semantic interoperability, which includes that context is provided when data are exported, as well as the meaning-preserving flow of information between different systems within and between different institutions, jurisdictions, and language groups [13].

## 3. Glossary

The focus of this paper is a thorough elucidation of tools, formalisms, and resources committed to the discipline of applied ontology [16] and particular methodologies for building, deploying, and evaluating them, which adhere to agreed-upon methods for encoding meaning, representing a domain, and creating semantic interoperability in healthcare and biomedical research.

For decades, the problem of standardizing healthcare data has been addressed by domain experts, informaticians, linguists, terminologists, AI experts, librarians, philosophers, and computer scientists. The extensive literature resulting from this effort itself uses different, partly contradicting expressions with the same meaning and, even worse, the same expressions with different meanings. Here, we have agreed on a consistent use of expressions throughout this paper. The glossary provided in Table 3 is fundamental for the subsequent deliberations in this paper.

## 4. The Role of Semantic Standards and Specifications

Based on the technical terms introduced in Table 3, this section elaborates on commonly accepted semantic standards or specifications [21], focusing on the following:

- Terminologies;
- Ontologies;
- Information models;
- Detailed clinical models;
- Process and guideline models.

We provide an overview and highlight current problems, which will be followed at the end of this White Paper by a list of recommendations.

### 4.1. Terminologies

Written and spoken communication in healthcare and biomedical research rely on natural language expressions (words, phrases, and idioms of various sorts). Technical subdomains have their own sublanguages. Because these languages and sublanguages are a product of evolutionary processes, numerous dialects have arisen and are progressively evolving in light of the need to keep up with the progress of science and technology. This causes problems for the development of controlled vocabularies or terminologies of the sort we are focusing on here. It also highlights a conflict between the need for the use of standardized language versus the need to accommodate the pressures of time and understandability dictated by the contexts under which language is used, and documents are produced in the context of healthcare.

Pressures of time and understandability dictate that clinical terminology must be both dynamic and compact. Its compactness has two major consequences, which matter whenever machine processing comes into play. On the one hand, it favors short forms like acronyms and abbreviations, which are difficult to expand and disambiguate. On the other hand, clinical utterances have to be interpreted in discourse contexts (e.g., whether a medical procedure has been performed or scheduled, whether a drug was prescribed or administered), which are often not explicit. Both of these pressures lead to shortfalls in standardization.

Among healthcare professionals, there is still a strong need to produce unconstrained free text, despite an increasing tendency towards structured documentation resulting from the use of EHRs. The proportion of ‘structured entry’ vs. ‘unstructured data entry’ carried out in EHRs by typing, dictation, and either transcription or speech recognition varies between jurisdictions, institutions, clinical specialties, and user groups.

However, even structured data acquisition struggles with human language issues. For example, drop-down menu entries (e.g., “normal” or “under control”) are understandable only in the context of data acquisition, which might be documentation of blood pressure, glucose level, etc.

Any use of the EHR data that requires further data processing will fail if the human language terms surfacing in the user interface—either because they form parts of value sets used in structured data entry or because they occur in free text data entry—are not formally related to an underlying terminological reference. These reference terminologies should provide codes as language-neutral representational units (RUs), which have the same meanings across all contexts of use. This should be guaranteed using self-explanatory labels attached to each code, together with definitions and accompanying comments providing indications of scope and examples of use [22].

Thesauri are a special kind of terminology resource. They comprise terms together with collections of (quasi-)synonyms. WordNet is perhaps the most comprehensive thesaurus artifact, but it is confined to common terms of natural language and has little coverage of technical terms from fields such as biomedicine [23].

The origins of thesauri lie in the need for library scientists to index the topics of books and articles for retrieval by users of literature databases such as MEDLINE. Such indexes are constructed hierarchically based on the subtopic relation so that an index entry for ‘cancer documentation’, for instance, might be arranged in the hierarchy under both ‘cancer’ and ‘disease documentation’.

Thesauri developed specifically for the medical domain suffer under the rapid evolution of technical language and the various preferences of different user groups, so their synonym coverage, too, is always insufficient, for example, lagging in their incorporation of new trade names for drugs. Despite the important role of medical thesauri like the medical subject headings (MeSH) [24] for literature retrieval and the UMLS Metathesaurus [25] for a broad range of applications, thesauri, and related artifacts cannot fully support interoperability between terminologies.

Such interoperability can be achieved by means of language-neutral representational units (RUs), each of which is defined in a context-neutral way. RUs of this sort are indispensable because the terms employed in EHRs and other information systems may vary in their meanings along many different dimensions. For example, the same clinical term from the same user interface terminology may need to be mapped to different RUs depending on the context of use in the system. Variation can arise because of hospital-specific practice in the use of terms or in the training of EHR data entry personnel. To see the problems that can arise, consider the set of terms mandated for use in the collection of data on race by the US Office of Management and Budget (OMB). Data collected using these terms are expected to be aggregated without concern about ambiguity of meaning. However, some data collection processes allow the respondent to choose more than one race, while others allow the respondent to choose only a single race and supplement the OMB categories with a “multiple race” category. The result is that a respondent who selects ‘Black/African American’ under the former scenario indicates some black ancestry, while a respondent who selects ‘Black/African American’ in the second scenario indicates only black ancestry. In each scenario, the same term from the same user interface terminology indicates a different RU.

To resolve such problems, terms used in user interfaces (often referred to as “value sets”) need to be mapped by the curators of the corresponding EHR resources to corresponding RUs in reference terminologies. At the same time, these RUs should be exploited to provide automatic guidance to users, for example, by ensuring that an acronym such as “RTA” is related appropriately to “road traffic accident” or “renal tubular acidosis”.

The role of patients as users of EHR systems (especially their potential to enter information about current complaints, drugs, and past and family history) is largely unexplored. User interfaces for patients need to be supported by health consumer vocabularies as a special type of user interface terminologies. Currently, the abundance of short forms in EHRs makes them mostly intractable for patients [26].

### 4.2. Formal Ontologies

This ordering of terms of a domain by subtopic brings advantages in helping humans gain access to needed textual information, as explained for thesauri, but it does not support the sort of reasoning that is required for many types of information-driven biomedical research. For this, the sort of hierarchical organization that is required must be based on what is called subsumption (or ‘is-a’) hierarchies, which allow information at lower (more granular) levels, in reality, to be aggregated upwards. If we know that some given data relates to an instance of X, and we know that Y is a term at a higher level than X in the is-a hierarchy, then we know that the data also relate to an instance of Y.

In the era of information-driven patient care and clinical research, therefore, we need not only terminologies based on language-neutral, precisely defined RUs that refer unambiguously to clinical entities (patients, body parts, procedures, disorders, drugs, and so forth) but also knowledge organizing systems organized hierarchically on the basis of subsumption. To this end, we also need the right sorts of principled, computer-tractable formalisms that are able to reason with data expressed using RUs of the given sort. This is the rationale for formal ontologies, which provide descriptions of the entities of a domain in a formal language that supports subsumption-based reasoning. We have seen a remarkable evolution of computable ontologies as annotation tools and representational artifacts in biology, spearheaded by the Gene Ontology [27], followed by other bio-ontologies, further aggregated by the Open Biological and Biomedical Ontology (OBO) Foundry [28]. In the area of healthcare, ontological principles have been increasingly incorporated into the large ontology-based clinical terminology SNOMED CT (see Box 4), which claims to provide semantically precise identifiers to represent the whole breadth of the EHR. These identifiers (SNOMED CT concept IDs) are the referents of the RUs in the terminology (and, by extension, of the associated codes in the EHR system and of related expressions in patient notes and so forth).

Consider, for example, patient Norville Rogers, who is a referent of the expression “Mr. Norville Rogers” in a doctor’s letter as well as of the patient id “#1234567” in some information system. In the same way, his diabetes is a referent of code “44054006”, and his retinopathy a referent of the code “4855003” in SNOMED CT. Formal ontologies support a clear-cut distinction between individuals (Norville, his diabetes, the Invokamet tablet he took this morning, his diabetic retinopathy) and types (homosapiens, type 2 diabetes mellitus, canagliflozin/metformin product, diabetic retinopathy). Ontologies provide, in addition, formal axioms, such as the following: “all instances of the pharma product canaglifozin/metformin have the substances canagliflozin and metformin as active ingredients”.

Terminology based on formal-ontological principles often produces linkages among drug and diseases that may appear tangential but are nevertheless legitimate and accurate. For example, a drug–drug interaction rule for metformin (SNOMED CT code 372567009) fires also if the prescription is Invokamet, given that Invokamet is linked to the SNOMED CT product code 714779001, which is, again, linked to the substance code 372567009 via the formal relation “has active ingredient”. Similarly, given that Norville’s retinopathy is referred to by the code 44054006 (diabetic retinopathy) it would also be retrieved by querying for a disorder of the eye (371405004) as well as a diabetic complication (74627003), by means of taxonomic subsumption.

### 4.3. Detailed Clinical Models

Reference terminologies and ontologies (see Table 3) are not assumed to interface directly with the end user, who interacts with them via interface terms, either by having clinical texts analyzed by NLP systems (whose performance will depend on the coverage of the language in the text by interface terms provided by an interface terminology linked to the reference terminology) or by structured input based on detailed clinical models (DCMs) to which terminologies are bound.

In order to prevent data silos, several standards for clinical models and their specifications have been proposed (e.g., openEHR, HL7 CDA, EN13606, CIMI, HL-7-FHIR), which, even if they are well structured, are buried in proprietary and non-interoperable formats. However, the adoption of such standards by manufacturers of clinical information systems has been low. What is important for our purposes here is that the use of the detailed clinical model (see Box 5) standards will alone provide no guarantee for semantic interoperability. Even if relevant elements in such standards are bound to standardized terminologies, so-called isosemantic models emerge [29], i.e., models representing the same content, although the models themselves are different. Undetected isosemantic models create problems for querying data, for example, leading to false negatives from a query that takes into account only one of the models. Good governance and interlinked communities are expected to overcome the isosemantic problem.

The tree structure of clinical models often suggests some parallels with ontologies. However, tree-like structures in clinical models mostly correspond to aggregation hierarchies of information entities, in contrast to the specialization hierarchies that characterize ontologies. Whereas ontologies express and define what is universally true for all members of a class, clinical models express factual or hypothetical statements about the individuals who are the primary referents of the clinical information.

The field of clinical information models has seen a remarkable dynamic during recent years due to the introduction of the new HL7 standard FHIR (see Box 6), pushed by a number of high-profile players in the healthcare informatics field.

### 4.4. Guideline and Process Models

Clinical guidelines and protocols are sets of rules to assist healthcare decisions under specific circumstances. They provide instructions on which tests to order or which services to provide. Clinical guidelines and protocols are commonly published as structured free text. The vision of adapting tools and resources from the planning and scheduling community to make clinical guidelines machine-processable has given rise to considerable research among computer scientists [32]. A number of formal languages for symbolic guideline representation have been developed, including the Arden Syntax GLIF, PROforma, and GDL. Unfortunately, there has been very little practical use of these formalisms. Much of this is due to the interoperability gap between clinical models and guideline specifications, sometimes known as the ‘VMR problem’, i.e., a standardized model of querying EHR data. However, it may also be connected with the fact that there are problems with the guidelines themselves, some of which are connected with the phenomenon of guideline proliferation. In November 2015, the National Guideline Clearinghouse [33] claimed that there were 486 guidelines for “Hypertension”, 517 guidelines for “Heart Failure”, and 129 guidelines for “Atrial Fibrillation”.

One line of research addressing guideline management in healthcare has taken its lead from workflow representation approaches developed in industry, for example, from the business process management (BPM) [34], the business entity lifecycle (BEL) [35], and the case management [36] approaches. There is some promise, particularly in the latter, which makes the assumption that business processes are by their nature full of exceptions and that any formal definition is likely to need adjustment during execution. It may be that formalisms such as Arden Syntax [37] and GLIF [38] will find new life in expressing decision rules within larger formalized case plans. Relevant standards include the OMG BPMN [39], CMMN [40], and DMN [41] standards. However, there is yet little experience with the case-based workflow approach in healthcare.

### 4.5. Interfaces or Mappings between Different Types of Standards and Specifications

The parallel use of different standards and specifications requires the definition of interfaces or mappings. Such user interfaces or mappings are, of necessity, expensive and difficult to maintain since the targets on either side will be developing independently of each other. In the following, we refer to the artifacts introduced and defined in Table 3, specifically reference terminologies, user interface terminologies, aggregation terminologies, clinical guideline specifications, and process models.

#### 4.5.1. Interfaces or Mappings between Reference Terminologies and User Interface Terminologies

User interface terminologies are mostly built in a community-driven, language-specific, bottom-up fashion. Examples are consumer health vocabularies [42] as well as many so-called purpose-specific “value sets” that are connected to reference terminologies, e.g., the epSOS Master Value Set Catalogue (epSOS MVC) [43], providing interface terms in several European languages linked to international terminology standards. User interface terminologies receive their semantic import by linking to reference terminologies/ontologies. The main problem to be faced is how to deal with the ambiguity of terms in the user interface terminology. This means that a single interface term may be mapped to different RUs. Especially short forms like acronyms tend to be highly ambiguous. Interface terminologies should therefore provide context information regarding the meaning of terms in different user groups and domain views.

#### 4.5.2. Interfaces or Mappings between Reference Terminologies and Ontologies

Ideally, ontologies would serve as the basis for reference terminologies such as SNOMED CT [44] so that the latter would be, in effect, an extension of the former. To the extent that this is the case, an interface between the two is unnecessary since interfacing is achieved through the sharing of codes. Bringing about a situation where SNOMED CT would itself be restructured in such a way as to constitute an extension of a coherently developed formal ontology is, we believe, something that can be achieved in an incremental way, and initial steps are indeed already underway. However, problems will still arise to the extent that reference terminologies have thesaurus-type features or exploit cognition- or natural language-based informal semantics, incompatible with the precepts of formal ontology. The mapping between reference terminologies and ontologies is also complicated wherever a RU from a terminology does not refer to a clinical entity type but is rather a matter of “epistemic intrusion” [45], as in “suspected pregnancy” or “missing ligand”. The latter does not refer to special sorts of entities but rather to states of knowledge on the part of a user [46].

#### 4.5.3. Interfaces or Mappings between Ontologies and Aggregation Terminologies

Aggregation terminologies like the WHO classifications have their own construction principles, which grow out of their original statistical rationale [47]. The goal is to achieve classifications, which, on any given taxonomic level within the hierarchy, are jointly exhaustive and mutually disjoint. Thus, the principles are designed (a) to ensure that the instances of all these classes sum to 100% of the instances of their common parent class and (b) to guarantee the mutual disjointness of the classes identified on any given level. This requires constructs like exclusion rules and residual classes (“Other New Zealander”, “Other mycoses, not elsewhere classified”, etc.), which cause problems for the stability of information conveyed using these classifications since their scope may change from one version to the next [48]. Despite different delineations, those classes often have the same labels as in reference terminologies and ontologies (for example, in ICD, but not in SNOMED CT, in which “Diabetes mellitus” excludes occurrences of this disease in pregnancy). This is a source of error that has often been unaccounted for, as terminology mapping based on lexical criteria is still common practice. The interface between ontologies and aggregation terminologies is, therefore, more complex than commonly supposed; an increasingly accepted approach is to represent the meanings of terms in aggregation terminologies as queries against ontologies [49], as the most faithful approach of expressing the intended meaning of RUs in aggregation terminologies.

#### 4.5.4. Interfaces between Ontologies and Clinical Models

The interface between ontologies and clinical models should ideally follow the line between ontology and epistemology. Whereas ontologies provide the meaning of well-defined domain-relevant entity types, clinical models provide the context of statements [50]. E.g., “open fracture of the left femur” would be expressed entirely by the ontology, whereas in “suspected fracture of the left femur”, “fracture of the left femur” would have separate referents because “suspected” does not specify the fracture but denotes the epistemic state of the author of this utterance. Since EHR information models and ontology-based terminologies like SNOMED CT have evolved independently, overlapping areas appear when they interplay, a well-known issue known as a “boundary problem” [51]. SNOMED CT, in particular, provides its own clinical model specification in the so-called context model [52]. Guidelines about what should be expressed in a terminology or ontology and what should be represented by an information model have been proposed but have found little acceptance. Alternatively, it has been suggested to root both the clinical world and the world of information in formal ontologies with well-defined ontology patterns that link statements, hypotheses, beliefs, etc., to clinical entity instances and types [53,54].

#### 4.5.5. Clinical Guideline Specification and Process Models

Despite some notable exceptions [55,56], semantic integration between ontologies and clinical guidelines has been largely neglected by both communities. A case study on how to model a simple clinical guideline rule related to heart failure within a formal-ontological framework proposed preliminary modeling patterns, which, however, require follow-up and further elaboration. The interface between clinical guidelines, clinical models, and ontologies presents numerous challenges [57]. Consistency of meaning and compatibility of representation must be assured, first, between guidelines themselves, particularly where these relate to the same or related disorders, in order to support effective empirical comparison of guidelines’ effectiveness. Nevertheless, consistency and compatibility also need to be assured between guidelines, clinical models, and ontologies in order to support effective computational management of guideline-relevant data. Guidelines may differ along a number of dimensions; for example, they may relate to clinical phenomena specified at different levels of generality (diabetes mellitus vs. insulin-dependent diabetes mellitus); they may relate to different sorts of assay information (raw measurements vs. “persistent highly elevated cholesterol”); or they may relate to different levels of granularity in specifying the actions to be performed in accordance with the guidelines. Formal means to determine the equivalence of meaning between expressions are needed (e.g., confirmed + hypertension vs. “confirmed hypertension”). Regarding process models, a preliminary alignment effort with an ontological upper level could benefit from accurate text definitions in a process model [58].

## 5. Human–Computer Interaction and Usability in EHR System Design

In the past decades, human–computer interaction paradigms have undergone an impressive evolution from the first graphical user interfaces and pointing devices to the current state of the art in touchscreen and voice interaction with mobile devices. Usability is defined as the capability of a software product to be understood, learned, and used, and all of this in a way that is attractive to the user (ISO/IEC 9126–1 (2001)) [59]. User interfaces in EHR systems, unfortunately, have not kept pace with what today’s users are accustomed to in their tablets and smartphones. The lack of attractiveness and intuitiveness of EHR user interfaces are assumed to have a strong impact on data quality and data recording time, as well as on patient safety and quality of care. These problems also increase the costs of training and retraining involved when new or replacement EHR systems are installed.

For many years, vendors insisted that usability was a subjective and unmeasurable concept. Taking a page from the usability literature, vendors argued that usability is dependent on the following:

1. The training and skill of the users;
2. The implementation of specific systems in specific settings;
3. The history of human interface technology used in any setting and by any user;
4. The relationship of a specific system to the other IT systems with which it must interact;
5. The physical environment (e.g., lighting, noise levels, and quality of display screens).

Other usability factors deserving mention are the frequency and degree of changes made by the host organization and by the vendor, as well as the degree of data interoperability with other IT systems in use in a given institution.

It is clear that all of these factors influence usability, often profoundly. However, none of them should be allowed to obscure the reality that usability is intimately dependent on the design of the system. Moreover, the fact that these factors play a role does not imply that usability is not measurable. Indeed, there are well-documented scientific methods for measuring usability, including measures that incorporate and acknowledge the other mentioned factors affecting use [60–63].

Testing usability (and iteratively improving usability based on the results of such testing) is an expensive, extensive, and ongoing process. Human interface technology vendors have, until recently, defended their lack of focused attention on usability by reiterating the mantra that usability is subjective or unmeasurable.

However, in the meantime, the frequent complaints by clinicians about clunky, slow, and unfriendly systems have reached the point where they can no longer be denied, and blaming physicians as hopeless technophobes is no longer a viable strategy. All vendors now pay homage to the importance of usability, although their level of understanding and commitment to the principles of user-centered design is highly variable. It is our view that usability must be built into an EHR system from the beginning. As a thought experiment, consider automobile safety. No one would deny that a car’s performance and braking ability are influenced by road conditions, the driver’s skill, and alertness. Yet, it would be absurd to insist that basic automobile design decisions do not seriously affect a car’s stability, safety, and braking effectiveness or to insist that there is no way of seriously studying the effects of car design on these and related factors. Some EHR vendors have claimed that there is only scant proof of the relationship between usability and safety. At the same time, and apparently, without irony, several vendors also note they have employed usability experts and that their own tests find their systems to be very usable [64–67].

One cannot test an EHR in one environment with a limited set of clinicians and then call the testing finished. True tests involve multiple, heterogeneous environments with scores, or even thousands, of clinicians and staff across the entire spectrum of those who will be called upon to use the system. Moreover, and more importantly, improvement and testing of usability is never finished. The systems themselves will be called upon to interact with other IT systems that are constantly changing and to interact with new environments, for example, patients with a new set of diseases, clinicians with different backgrounds, new equipment, and new requirements. The EHR vendor association (EHRA), a subsection of the Health Information Management Systems Society (HIMSS), admits that usability is the primary challenge and the major barrier to the wider acceptance of EHR systems.

### Clinical Decision Support

We tend to regard computerized clinical decision support (CDS) as one of the major benefits of EHR technology [68], but CDS is everywhere hated for the vast number of false alerts it generates [69]. The logic underlying the ways in which CDS compiles and uses evidence is often more dubious than generally understood. For example, the data on which CDS information is based require clinical trial sample selection and protocols that restrict subjects to patients with only one disease and one medication. This restriction is good for science (for isolating empirically detectable regularities). However, it is useless for application to the normal run of patients in a real-world healthcare institution.

In addition, because of the limits of EHR data standards and interoperability, CDS systems cannot mine the vast oceans of information that would otherwise be available to bring about progressive refinements in the results they yield. The necessary nuanced understanding of the multivariate issues involved in real-world cases—of a sort that can be conveyed to the computer—is usually impossible. What do we know about the interactions of the 4000–5000 drugs in the average formulary? How can we match that almost infinite matrix with the additional constraints brought on where patients have compromised kidney, liver, and cardiovascular functions?

CDS is presented without the context of its application and knowledge of its end users. This means that the alerts may differ from ward to ward, service to service. Interns and many residents, who rotate every thirty days, often depend on dosage alerts, order sets, or drug–drug interaction (DDI) alerts when confronted with unfamiliar medications. Because the range of permitted dosages, and even the existence of any alerts, can vary from service to service and from hospital to hospital, residents often prescribe with the expectation of a safety net comprised of warnings and alerts. Alas, the net may be missing or configured for very different purposes. Medication orders are entered with the false belief that dangerous doses or combinations are systematically flagged.

The critical issue raised here is that of information presentation. This issue has not been sufficiently addressed by EHR systems. Information presentation includes (i) how existing information is visualized (presented to the user) on the one hand and (ii) how (under what real-world conditions) data entry is performed. Current systems flood the user with largely monotonous, structured, and unstructured, often redundant data and documents, ordered only by date and document type. Preselection, summarization, and prioritization would reduce the risk of clinicians missing important information due to time pressure.

Regarding support for data entry, current EHR systems fall short in shaping data entry options in a way that prioritizes meaningful information, with the effect of notorious cases of the sort cited in Section 2.1, Box 2. More and more consumer applications, for example, on our mobile devices, have incorporated frequency-based approaches, where the user sees those values first that they have used most frequently in the past. In addition, statistical associations exploit large corpora of existing data. The paradigms of big data (see Box 7) and learning health systems (see Box 3) might ground a new generation of systems that adapt to the user by constantly analyzing their interaction with IT systems, as well as the content they produce and query.

## 6. Recommendations

Considering the benefits provided by intelligent clinical information processing based on ontologies in comparison to alternative approaches, priority should be granted by funding agencies. The main rationale is the quality of clinical data for healthcare and translational research. Such research projects should be multicentric and outcome-oriented, and they require sufficient resources for large-scale evaluation benchmarks. Annotator/coder agreement as a key indicator for the quality of human or machine use of ontologies and terminologies should be subject to continuous monitoring. Key performance indicators to measure the progress of investments in clinical terminologies and ontologies need to be developed. Modeling is not enough to convince influential stakeholders. Research programs might include periodic challenges similar to the i2b2 NLP challenges. The whole ontology value chain needs to be demonstrated to convince potential adopters.

Translational research is multiscale research; it spans from populations (macroscale) through organisms, body parts, cells, and proteins to small molecules (microscale). Ontology research, ontology engineering, and ontology integration must mirror this spectrum. Thus far, SNOMED CT, as a clinical terminology/ontology, addresses the macroscale primarily, whereas most successful bio-ontologies, such as the Gene or Protein Ontology [71–73], ChEBI [74], etc., address the microscale. Some biomedical ontologies, such as the Drug Ontology [75], incorporate phenomena at both scales. Ontology research for translational science must aim at developing strategies for multiscale ontology integration. So far, SNOMED CT is completely self-contained, i.e., it does not refer to any external resources. This is justified by legal reasons when used for patient documentation, but it falls short wherever multiscale integration is at stake.

A new paradigm for clinical computing has to be developed. Clinical data should be maximally explicit and self-explanatory [76]. ‘Maximally explicit’ means that each such repository should contain explicit reference to any and all the entities, including their interrelationships that must exist for an assertion encoded in the repository to be a faithful representation of the corresponding part of reality [77]. By ‘maximally self-explanatory’, we mean that the data in the repository should be presented in such a way that a researcher seeking to query the repository does not need to concern themselves with any idiosyncrasies of and between datasets, codes, and formats that were combined or used to build the repository. This requires that the vast range and types of information about single patients or patient cohorts can be retrieved by a declarative language for statements, questions, and answers. A simple answer-set semantics, closely aligned with natural language question-answering, would operate on hidden, ontology-based knowledge structures in clinical data warehouses [78]. Querying patient data also requires querying ontologies using description logic query and metaquery languages [79].

User interface terminologies that include lay terms are crucial for the patient’s role as an active EHR user. User interface terminologies need to cover elliptical metaphorical language (such as “sugar” for diabetes) as well as allow shallow and unspecific terms (“liver problem”) and modifiers for uncertainty (for example, use of terms such as “possible” and “likely”), but also all kinds of clinical jargon, typically found in clinical documents.

Ontology research and standardization should favor top-down standardization from a philosophically based top level like Basic Formal Ontology (BFO) [80], down to a level of approximately Ontology for General Medical Science (OGMS) [81]/BioTop [82], also including de facto standards such as the OBO Foundry ontologies. It should include ontology-based representations of the basic elements of information models, clinical process models, and guideline models.

A focus of applied ontology research should address best practices that show for selected purposes and requirements how ontology can support them. This should address existing knowledge organization systems (KOS) in a broad sense (including biomedical thesauri, nomenclatures, vocabularies, and classifications), with recommendations of what KOS and standards to keep and how to modify existing KOS and standards or develop new ones.

Up to now, few semantic standards in the EHR area have proceeded on any solid theoretical basis, regardless of whether we strictly look at de jure standards or also include de facto standards (see definitions in Table 3). Instead, many standards have grown out of round-table discussions trying to patch together piecemeal lowest-common-denominator mashups of existing data structures. This could be mitigated by establishing a semiformal description of an overall health computing architecture that places terminologies, information models, content models, guidelines, referent tracking, and portable querying into an overarching ontological framework. Without such a framework, most EHR standards work is likely to remain incoherent and disconnected. Terminology remains difficult while the industry assumption that all terminologies look like SNOMED CT or ICD persists, and the related assumption that anyone who is not using SNOMED CT is irrelevant.

We must investigate how the needed standards can be created, tested, and adopted more rapidly, driven by good practice examples from successful standards development work. New semantic standards should focus on cross-scale issues in the context of translational science and personalized health. Forces should be joined—also at an international scale—to improve standards, especially the ones that are large, increasingly adopted, and largely improvable. The prime candidate for this is SNOMED CT, which could benefit immensely from a better alignment with existing ontological and terminological resources, formalisms, and principles. This is not something that might be delivered by SNOMED International working alone. It will require a massive international and interdisciplinary effort by the biomedical semantics community together with the curators of SNOMED CT. It will also require the elaboration of a distributed, international governance model for terminological/ontological standards.

Although SNOMED CT is still plagued by numerous issues that derive from its legacy and ontologically half-hearted design decisions [83,84], the authors of this paper believe that an incremental redesign based on formal-ontological principles is possible. There are encouraging yet under-resourced initiatives within SNOMED International. Joint efforts of the biomedical semantics community should give priority to (i) the alignment with formalisms and languages of the Applied Ontology and Semantic Web communities; (ii) the identification of underspecified content, which requires elucidation by textual scope notes and/or formal definitions to be unambiguously understandable; (iii) the redesign of content areas that contradict basic notions of formal ontologies, particularly the separation between clinical entities and informational entities (extending to a principled representation of the ontology/information model interface); (iv) the correction of logically flawed modeling, e.g., regarding negation; (v) a clean but nevertheless usable solution for the complex disease/disorder/findings, making it compatible with related ontologies; (vi) separating SNOMED CT’s ontology/reference terminology view from the interface terminology/value set view [85].

Each EHR system should allow the export of data in optimal quality (see Box 1). A first step towards achieving this is the mandated use of a standard for terminologies and for information models (e.g., SNOMED CT, CDA, and USCDI), ideally within an overarching ontological framework, which also includes ontology-based mappings between different genres of terminologies, such as between SNOMED CT and ICD. But much more than this is required.

A focus needs to be placed on interconnections between human language/interface terms and reference terminologies/ontologies. This should be mediated by analyses of clinical processes and of real-life text corpora (clinical texts, scientific texts, guidelines, and protocols). The multiplicity of surface expressions to encode identical meanings needs to be harvested. This requires continuous end-user involvement. Crowdsourcing mechanisms and related incentive models need to be developed and validated.

Postcoordination mechanisms within ontologies should be increasingly used, supported by easy-to-understand composition mechanisms, the usefulness of postcoordination in terms of revealing equivalences (of syntactically different structures) by machine reasoning must be demonstrated, as well as the use of postcoordination in data querying.

Data quality requires the management of data provenance, originator role, currency, evidence, and related strength/certainty. Cross-validation of data quality should use multisource evidence and mutually supportive evidence, i.e., the scrutiny of internal and external consistency.

The improvement of data quality should include both preventive and post hoc measures, i.e., data cleansing.

A typical post hoc approach is the processing of unstructured or semistructured data using a combination of state-of-the-art natural language processing (NLP) technologies fed by high-quality and high-coverage interface terminologies that are connected to a clinical ontology, providing representational patterns for both clinical and information entities. The resulting semantic representation should be evaluated for correctness and expressiveness against a benchmark. There have been many such projects in the past, but no large-scale one that really exploited and demonstrated the power of ontology-based reasoning.

Regarding the fitness of current EHR systems, tasks must be identified where it can be improved with minimal disruption already now, simply by addressing the quality of the underlying terminological, ontological, and information model standards and specifications for data acquisition and sharing. One example is the improvement of ontology-based markup of clinical narratives. In contradistinction, other tasks need to be formulated that will require more radical changes to EHR systems in the future.

The fact that EHR systems so dramatically lag behind in usability when compared to similar artifacts in other spheres requires a series of actions. To quantify this problem, all of the following dimensions will need to be taken into account: waste of time and money, user dissatisfaction, suboptimal quality of care with implications also for patient safety, and missed opportunities in data reuse for purposes of research. More evidence along all these dimensions will need to be documented through qualitative and quantitative research. Based on this evidence, a coordinated effort should then be mounted with the goal of calling forth changes in EHR vendor behavior regarding semantics and usability.

However, substantial effort is required to pressure EHR manufacturers to invest in customizable and user-friendly data entry tools which implement the most up-to-date usability paradigms, encompassing text or voice entry, if needed, together with graphical entry as modules for passive information recording by observation and instrumentation. This effort must include usability lab experiments as well as large-scale design thinking activities, as joint efforts between industry, academia, and user groups.

The most critical usability issue is data entry. Frequency-based shaping of data entry options need to be put in place, both based on the individual users’ past behavior and by exploiting “big data”. It seems obvious that ontologies can be used to constrain the range of possible entries, e.g., by constraining that only female patients experience menstruation or that only bones are the site of fractures. However, this needs to be demonstrated by good practice examples.

Frequency-based and ontology-based approaches are not mutually exclusive, and it is likely that some combination of them best supports usability, which should also be subject to systematic investigation.

Finally, new approaches to customized visualization should be developed and tested, e.g., content filtering, prioritization, and content summarization, with the goal of highlighting those pieces of information that are relevant in the current decision process, dependent on situational and user contexts.

## Figures and Tables

**Table 1.** Purposes and requirements for electronic health records (EHRs).

EHR Purposes	EHR Requirements

**“Classical”** • Communication and data sharing among health professionals and care providers • Ordering procedures • Ordering drugs • Computing of quality-of-care metrics (scopes: healthcare provider, geographic area, populations) • Obtaining approval from health insurer • Billing **“Advanced”** • Explaining conditions and treatments to patients • Discovery of incompatibilities and treatment errors • Identification and prevention of patient safety issues • Informing adherence to clinical guidelines • Automated decision support by automated reasoning using medical knowledge • Automated decision support by case-based reasoning using large EHR datasets • Discovery of possible improvements in treatment • Prediction of impending and future health problems • Extracting reliable phenotypic signatures for clinical genomics research • Candidate identification for clinical trials • Population-wide screening • Data analytics for research or business processes • Cross-EHR retrieval for cases for teaching • Question answering	• Ease of input, following current usability paradigms • Multimodal input (text, voice, images including scanned end handwritten documents, signals) • Typing error detection and correction • Context-dependent support for data entry • Personalized voice and text entry support • Direct input from devices • Coding from device input • Coding from images • Coding from provider notes (typed, handwritten, voice) • Terminology control and ambiguity detection at typing • Detecting all pieces of information that could be used for personal identification • Display and retrieval of information within or across individual EHRs • Personalized summary display with drill-down to detail • Syntactic and semantic interoperability with many different systems • Recording data provenance, i.e., internally or externally, device that captured data, etc. • Using fine-grained standards that support research • Using data standards that are employed by or compatible with outside data sources

**Table 2.** Frequent problems that often stand in the way of EHR use for translational research.

Problems Observed in Certain EHR Instances	Characteristics of These Problems

EHR data are inaccurate	EHR data contain too many errors—from carelessly written texts, copy-and-paste errors to clinical coding [8].
EHR data are difficult or impossible to interpret	Complete interpretation of EHR content requires hidden contexts to be made explicit.
EHR data tell an incomplete story	Missing data elements abound.
EHR data across organizations are inconsistent	Patients are treated in many places; different organizations’ EHR typically records different information on the same patient; even within the same organization and with the same EHR system, data on a given patient may be inconsistent because of different installed features or different levels of training.
EHR data are tilted toward the needs of billing and administration	Much data derives from coding of diagnoses and procedures for billing and exhibits even greater inaccuracy than EHR data proper. This is particularly problematic where insurance companies require a specific diagnosis to be present in order to pay for specific procedures, medications, or other treatments.
EHR data include too much free text	The vast majority of the patient’s story is told in narrative text, but natural language processing (NLP) technology is still too far from perfect for routine use. NLP is complex to set up, use, and maintain. To work well, NLP solutions need to be tailored to document types, domains, and sometimes specific healthcare providers. The quality of NLP solutions critically depends on large training resources, which are expensive to create and are often not available due to privacy concerns.
EHR data lack provenance	EHRs are constantly fed by external information systems (e.g., lab systems, connected devices), but they do not always indicate the provenance (source systems and organizations) of these data.
EHR data are too coarse-grained for research	Coding for billing is at the level of diagnosis categories, not fine-grained diagnoses. Besides imparting loss of information, heterogeneous sets of diseases or procedures are identified by the same code.
EHR data are derived from clinical care and lack of granularity for research purposes	Clinical care rarely matches the level of rigor in measurement, calibration, and data collection that is required for clinical research.

**Table 3.** Basic terms and definitions.

Term	Definition	Note

representational unit (RU)	A language-independent denotator that corresponds to a node in a terminology or ontology.	RUs are typically identified by a code and a human-readable label. Optionally, their meaning is described by scope notes and by textual and logic-based elucidations or definitions.
term	A linguistic expression (ranging from a single word to a phrase) that belongs to a domain-specific vocabulary in a given natural language.	We also distinguish labels from terms (see “label”).
label	An often artificially constructed term that aims to attach a maximum of unambiguous meaning to an RU.	For example, “Biopsy of head and neck structure” is an unambiguous label but might not be a term that would be commonly used by practitioners.
concept	No definition given because “concept” is used in numerous, partly contradictory senses [17]. Despite the immense popularity of this word, we recommend always using it with an attribute, e.g., “SNOMED CT concept”, in order to avoid ambiguous interpretations (in which case it is referencing an RU in the associated artifact).	According to the community, it is used in the sense of “entity of thought” (encompassing classes, binary relations, and individuals in SNOMED CT; classes and individuals in UMLS), “information template” (clinical model community), “unary predicate” (logic), “universal” (ontology).
class	A group of things that share some properties.	Linguistic expressions (terms, labels, scope notes, definitions), as well as formal axioms, describe the class and criteria for membership. Most RUs (nodes) in ontologies denote classes. Synonym: universal
individual	A single thing in a domain.	Individuals are members of classes.Synonym: particular.
terminology	An information artifact that includes a “set of designations belonging within a discipline”.	Normally, these designations are “terms”, i.e., units of human language, but codes might also be encompassed. Given the broad use of “terminology” in biomedical informatics as well as in biomedical science (beyond the common use of “terminology” in the terminology world), we recommend always using it together with qualifying adjectives.
reference terminology	A terminology that organizes RUs in a domain, with human readable, maximally self-explaining labels and potentially formal or textual definitions or scope notes.	Reference terminologies are uncommitted to any specific purpose. Their representational units are often named “concepts”, e.g., SNOMED CT concepts.
aggregation terminology	A terminology where RUs are systematically organized in single nonoverlapping hierarchies, enhanced by classification rules.	Aggregation terminologies are also known as classifications, e.g., the WHO classifications such as the International Classification of Diseases. Aggregation terminologies are meant for specific purposes like data aggregation and ordering. Synonym: classification.
user interface terminology	A terminology containing terms used in written and oral communication within specific contexts determined by language, dialect, application, and user groups.	User interface terminologies either lack semantic import altogether or acquire their necessary semantic import by linkage to reference terminologies/ontologies or aggregation terminologies. They are often ambiguous and, therefore, not just alternative labels. User interface terminologies obey less strict organizing principles; terms are grouped topically rather than ontologically.
thesaurus	An informal terminology that groups together words and terms according to similarity of meaning.	In biomedical informatics, there is a tendency to extend the meaning of the word "ontology" also to thesauri (e.g., MeSH, NCIt, UMLS), which we recommend to avoid.
formal ontology	A representational artifact, comprising a taxonomy as proper part, whose representational units are intended to designate some combination of universals, defined classes, and relations between them.	The use of the word “ontology” should be limited to carefully engineered, principled, and computable models of meaning. As such, ontologies can be described as formally founded reference terminologies. Thesauri (e.g., MeSH) or simple data models (i2b2) are not ontologies in our sense. We avoid the use of “ontology” in the sense of the Obrst semantic spectrum [18]. To enhance clarity, we recommend the use of this composed term “formal ontology”.
standard	An information artifact developed in community-driven consensus processes that specifies uniform criteria, methods, processes, and/or practices for a certain domain.	The term “standard” is also used in a broader sense for specifications that adopt a “de facto” standard status due to acceptance by a large public or market forces.The term “standard” may be applied (i) to single artifacts, which may be huge (e.g., SNOMED CT) or tiny (a single ISO13606 or openEHR archetype), (ii) processes for creating artifacts that connect into a larger whole, and (iii) shells that support the creation of (i) by (ii) by numerous distributed parties.
quasi-standard	A specification that is not the outcome of a standardization process but is nevertheless accepted by a larger community.	Due to their large acceptance, quasi-standards can be relatively safely referred to where real standards do not exist.Synonym: de facto standard
information model	An information artifact for a specific domain like healthcare, by which a bounded set of facts, assertions, and instructions are expressed to meet a specified requirement, typically that of an implementation.	Elements of an information model can be instantiated to create persistent and/or in-memory data.
reference model	A model that typically defines a logical model of data based on very generic entities (classes) and data types.	In the healthcare domain, reference models are often published as standards [19,20].
semantic interoperability	The ability of the flow of information between different components within the same or different systems or institutions to be meaning-preserving.	Semantic interoperability attracts growing attention in the area of biomedical semantics.
scale	The level of granularity in describing physical entities.	Macroscale: the patient—microscale: genes, proteins, etc.

## Data Availability

Not applicable.

## Box 1. Data quality.

Quality consists of the ability to achieve desirable objectives using legitimate means [2]. The characteristics of data quality in a clinical context were described as follows [3]: Accessibility: data are easy to obtain and legal to collect;

- Accuracy: values are correct and valid;
- Comprehensiveness: all data required for a given purpose are included;
- Consistency: the value of the data is reliable and the same across applications;
- Currency: the value of a datum is current for a specific point in time;
- Definitions: clear definitions assure that current and future users will know what the data means;
- Granularity: attributes and values are defined at the correct level of detail;
- Precision: data values are exact enough to support the application;
- Relevancy: data are meaningful to the performance of the application for which they are collected;
- Timeliness: timeliness is determined by how the data are being used.

Regarding scholarly data, the recently proposed FAIR Guiding Principles [4] have found broad acceptance. FAIR stands for Findable, Accessible, Interoperable, and Reusable.

## Box 2. Bad usability examples.

Emergency room physicians who wish to order an X-ray conducted in the radiology suite five steps from the emergency room must typically answer the question of how the patient will get to the radiology department. Sensible options are “wheelchair” and “bed”. We know of one EHR system in which the option “helicopter” is also provided. This option is meaningless, distracting, and creates the potential for avoidable data entry errors. Here, as in other cases, lists should have been tailored for the situation. This example illustrates how vocabulary issues (in this case: creating picklists of allowed values) can impact not merely the accuracy of data but also system usability, system interoperability (what is the receiving radiology system supposed to do with “helicopter”?), and data reusability (how is the patient-safety researcher who is studying the effect of patient transport on fall risk supposed to handle “helicopter” in her research?).

Another example is the need for EHR users to scroll past “fetal monitoring” reports on their way to find progress notes for an 83-year-old. Some EHRs cannot find ‘essential hypertension’ when the physician searches for ‘hypertension’.

## Box 3. Learning health systems.

Some classical problems in the current model of medical knowledge production, transfer, and application could be solved by what is referred to as a learning health system (LHS) [14,15]. An LHS is a system involving a close coupling between care delivery, research, and knowledge transfer, in which health information generated from patients within that system is continuously analyzed to create new knowledge and then transferred to the clinician to improve patient healthcare.

An LHS ideally should involve primary care as the first contact for most patients with health service and then follows them continuously. As such, they require the integration of information about a patient from several healthcare providers. Learning health systems can be developed using data mediation (local-as-view), in which a central model is used to formulate queries, which are then translated to be run locally in each data source; results are then aggregated centrally. A proof of concept of an international LHS involving primary care has been provided by the European TRANSFoRm project, which used the CDIM (clinical data integration model) ontology as a central model.

## Box 4. SNOMED CT.

SNOMED CT is the largest clinical terminology with about 350,000 concepts (representational units) to which more than 700,000 English terms are linked. It is maintained by the international standard development organization SNOMED International (former IHTSDO). Although SNOMED CT is distributed in a triple-like, relational form, its architecture incorporates some ontology principles, and an official conversion mechanism into the ontology language OWL-EL is available. SNOMED CT also supports postcoordination, a mechanism that allows the building of logical, well-structured expressions by combining existing SNOMED CT expressions to create terms with correspondingly more complex meanings. However, we are not aware of the significant use of postcoordination by health IT software developers.

In its current state, SNOMED CT increasingly fulfills the desideratum of providing a reference terminology underpinned by a formal ontology that would allow reasoning of the sort described in the text and flexibility of use when faced with new applications. Scrutiny reveals some issues with the way the terminology is structured that block plausible inferences. Despite an increasing agreement that domain ontologies should be guided by clear principles and ontology-based top-down standardization, the arrangement of SNOMED CT content still reflects its legacy in approaches, which need to be scrutinized against principles of good ontology design. There are still divergences in how to conceptualize clinically relevant conditions of a patient and in how to delineate body structures and regions.

SNOMED CT still has many concepts that lack definition and only gradually introduces definitional scope notes. E.g., terms like “adolescent”, “child”, “anxiety”, and “fear” lack elucidation, so their use might introduce unreliability since users will rely on their own intuition or they will refer to definitions from multiple external sources, which may not be mutually consistent. (For example, the WHO defines “adolescence”, in contradiction to most other sources, to include 10-year-old children). Part of the function of terminological standards and ontologies should be to provide clear and consistent definitions to guide the use of terms like this, which incorporate certain vagueness. Current efforts and content development strategies put in place by SNOMED International promise a move towards more ontological clarity in these respects.

If, as is the case with SNOMED CT, representational units and relations in an ontology are neither formally nor textually defined, this will lead to a situation where terms used in clinical texts and clinical models are connected by different users to different RUs within the ontology. Then, the usability of the ontology will be negatively affected by the inevitable high rates of disagreement between experts on which terminology code to employ in given contexts. The more an ontology grows in depth and coverage, the more consistency of representation is negatively affected in this way. Continuous efforts to fix under-specifications become necessary, as well as detailed guidelines for annotation and binding to clinical models, tooling, and associated educational resources. All of these, in turn, need to be developed in a consistent fashion.

## Box 5. Detailed clinical models.

Detailed clinical models (DCMs, ISO/TS 13972:2015) are an attempt to specify the data elements relevant for each type of clinical entity, including the relevant attributes and values and the relations needed to convey the underlying clinical reality in a way that is understandable to both clinical domain experts and modelers. The approach, which amounts to constraining an information model (see Table 3) so as to create a kind of template model for recording information, is also known under the following headings: archetypes (openEHR, ISO 13606–2), templates (ISO 10303–1:1994), clinical element models (Intermountain Healthcare), and so forth. These approaches constitute an important class of specifications designed to support the entry of structured information into EHRs by ensuring that the clinician does not fail to document important information. They also serve to organize the EHR content in databases in such a way as to enhance retrieval. Unfortunately, for any given domain entity—such as ‘liver function test result’ or ‘medication order’—there exist numerous ‘models’. These entities themselves are not described ontologically since the job of the clinical models is merely to indicate the associated information structures [30].

## Box 6. HL7 FHIR (Fast Healthcare Interoperability Resources).

The new HL7 standard Fast Healthcare Interoperability Resources (FHIR) [31] provides a granular way to exchange data without the rigid workflow of traditional HL7, using a RESTful style approach. FHIR focuses on providing clinical models that offer solutions to frequently occurring problems, for example, problems that arise in connection with information exchange between different EHRs. Its clinical models are bound to standardized terminologies and/or ontologies.

FHIR is an open standard with a high alignment with the Semantic Web, representing a new EHR modeling paradigm based on interoperable building blocks named “resources”. A resource is a small data model that defines a set of properties describing certain aspects of a domain, e.g., condition, medication, and observation. There are approximately one hundred resources classified into six categories, and each is uniquely identified with a Uniform Resource Identifier (URI). Examples are patient, practitioner, medication order, and observation. FHIR resources can be serialized in JSON and XML, and there exists a draft RDF representation. Although FHIR was not designed with the Semantic Web and RDF in mind, FHIR resources and links between them align well. HL7, in collaboration with W3C, has proposed an RDF representation for FHIR aiming at improving semantic interoperability with other standards. Due to the nature of RDF and the structure of FHIR, this HL7 FHIR RDF representation focuses on representing the structure of a resource rather than the semantics of the corresponding content.

## Box 7. Big data.

Big data is sometimes seen as a phenomenon that overrules classical symbolic data and knowledge representation, with the argument that the overwhelming volume of heterogeneous data on the web constitutes the ideal knowledge base once the appropriate tools are used to draw out its rich potential. Human-made models, in contrast, are seen as lagging behind what big data makes possible, so they will eventually be completely dropped. Applied to the EHR context, such arguments could be used to challenge the field of biomedical semantics as well as the efforts invested in semantic and terminological resources altogether. It is crucial for the translational medicine community to reject such visions based on empirically well-grounded counterarguments. The main argument is that precision medicine requires precision data. This means that decision support and retrieval cannot depend on large-scale black box-style analytics of noisy, imprecise data. Big data can certainly be an ally in constructing precision resources. We hypothesize that the abstraction of the individuality of natural language utterances into structured and semantically normalized information facilitates the use of statistical data analytics in order to distill new knowledge out of textual data from biomedical research and clinical routine. Several approaches of NLP technologies on clinical texts depend on linguistic and terminological resources, the creation of which is labor-intensive. Here, it is sensible to assume that big data methods can prove useful. Examples include the learning of hierarchical relationships, the grouping of synonymous terms, and the disambiguation of homonyms, e.g., by new approaches like neural network-based deep learning [70].
